# Explaining health care providers’ perceptions about the integration of palliative care with primary health care; a qualitative study

**DOI:** 10.1186/s12875-022-01835-3

**Published:** 2022-09-07

**Authors:** Suzanne Hojjat-Assari, Maryam Rassouli, Vahid Kaveh, Heshmatolah Heydari

**Affiliations:** 1 Immunohematologist, French Institute of Research and High Education (IFRES-INT), Paris, France; 2 Cancer Research Center, Shahid Beheshti University of Medical Sciences, Tehran, Iran; 3 Depertment of Medical Oncology and Hematology, Iran University of Medical Sciences , Tehran, Iran; 4 School of Nursing and Midwifery, Social Determinants of Health Research Center, Lorestan University of Medical Sciences, Khorramabad, Iran; 5 French Institute of Research and High Education (IFRES-INT), Paris, France

**Keywords:** Community health care, Home health care, Advanced cancer, Palliative care Terminally ill, Qualitative study

## Abstract

**Background:**

Easy access to palliative care is one of the basic needs of cancer patients, and this can be achieved by providing such services at the community level. One approach to provide community-based palliative care is to integrate it with primary health care (PHC). Considering the antiquity and extension of the PHC system in Iran and the importance of being aware of stakeholders’ views in order to integrate a palliative care provision model into a country’s health care system, we aimed to explain health care providers’ perception of the integration of palliative care with PHC.

**Methods:**

The present qualitative research was conducted using the conventional content analysis method in Iran from October 2016 to July 2020. The participants of the study included the stakeholders involved in providing palliative care to cancer patients, as well as PHC system experts. The participants were selected purposefully using the snowball sampling method. Data were collected through holding 21 semi-structured interviews and one focused group session and analyzed based on the method proposed by Lundman and Graneheim.

**Results:**

Qualitative data analysis revealed three main categories and ten subcategories. The main categories included the health system’s structure as an opportunity (with the subcategories of employing the network system for providing health services, establishment of a referral system, and establishment of the family physician program and manpower diversity), requirements (with the subcategories of the position of home care centers and their relationship with PHC, opioid use management, equipment management, financial support, and legal issues), and outcomes (with the subcategories of facilitated access to services and good death).

**Conclusion:**

Iran’s health system possesses adequate infrastructure for providing palliative care to cancer patients within the context of PHC. Beside available opportunities, there are also problems that need to be resolved so that families can meet their patients’ care needs and provide them with an easy death by having access to home-based palliative care.

**Supplementary Information:**

The online version contains supplementary material available at 10.1186/s12875-022-01835-3.

## Background

Cancer is known as the second leading cause of death worldwide [1]. The International Agency for Research on Cancer estimated a global cancer incidence rate of 19.3 million cases with 10 million deaths in 2020 [2]. Cancer is also the second leading cause of death in Iran [3]. The total number of cancer patients in Iran was 110,115 in 2018, causing 55,785 deaths in the same year [4].

Palliative care, as one of cancer patients’ needs, has been recognized by the World Health Organization (WHO) as a warrant for improving the quality of life of end-stage cancer patients [5]. Palliative care is a comprehensive care method that considers the whole existence of humans, including their physical, psychological, social, and spiritual dimensions [7]. This type of care not only helps patients live their lives until the last moment, but also supports patients’ families during the disease course, at the time of the patient’s death, and even after death, helping them better and more calmly and peacefully accept the phenomenon [6].

According to a report by the WHO, palliative care and primary health care (PHC) have a number of common principles such as continuous care, social responsiveness, respecting patients’ values, and paying attention to the patient’s care needs in the presence of his/her family members. So, the WHO recommended in 2016 that in order to achieve the goals of sustainable development and universal health coverage (UHC), health systems must integrate community-based palliative care into PHC programs [6] so that these services can be provided to patients and families by the clinicians who are in close contact with them [7]. According to Astana Declaration, PHC is the foundation of a sustainable health system for UHC and health-related Sustainable Development Goals (SDG), allowing to meet health care needs of people during their lifetimes via delivering comprehensive preventive, premonitory, curative, and rehabilitative services, as well as palliative care [8].

The advantages of community-based palliative care include the facilitation of patients’ access to health care [9], promotion of the quality of life of patients and caregivers, reduction of hospitalization length [10] and the rate of referral to emergency departments [11], and finally an increase in the number of at-home peaceful deaths [12].

Iran’s health system follows a hierarchical referral network, and its services to the community are provided in the form of PHC. In this structure, comprehensive urban and rural health centers are responsible to deliver health services to the people of a specific region [13]. Nevertheless, palliative care, as a novel care approach, has no place in this structure. In fact, such services are provided to the community by private and charity organizations [14]. It is obvious that patients and their families become baffled and experience high distress, confusion, and agony when they encounter advanced cancer. Given the desire of patients to receive at-home health care [15] and the appropriate structure of PHC in Iran, it seems that the integration of palliative care into this structure can greatly help cancer patients and their families receive their required services.

To establish health services in a specific field and to design a care provision model for these services, that field must be well-recognized.

This requires knowing the context in terms of facilities, opportunities, and barriers from the perspective of health care providers. This goal can be achieved by qualitative research. Therefore, this study was designed to explain health care providers’ perception of the integration of palliative care with PHC.

## Methods

### Design

This was a qualitative study performed via the conventional content analysis approach in Iran from October 2016 to July 2020.

### Participants

Participants were selected through purposeful sampling and included oncologists, palliative care specialists, general practitioners, nurses, psychologists, social workers, and PHC system experts who were selected applying a snowball approach by referring to target colleges, home health care centers, comprehensive health care centers, and cancer departments. Sampling continued until reaching data saturation (i.e., the time at which no new codes were extracted from interviews) [16]. In addition to individual interviews, a focus group was held with community-based palliative care experts to enrich the data. In order to be included in the study, health care providers had to have at least one year of experience in providing home- and community-based palliative care to patients. Policymakers should have had roles in policymaking for cancer patients and their families regarding home- and community-based palliative care services for at least three months. Inability or unwillingness to participate in the study were regarded as exclusion criteria.

### Data collection

The main tool of data collection included in-depth semi-structured face-to-face interviews. The main probing question for directing the interview varied according to the interviewee’s knowledge, expertise, and experience. For data collection, cancer patients, families, and care providers were identified and selected by referring to the centers and hospitals providing home-based palliative care services. After participant identification and qualification, the time and place of the interview were specified. The duration of each interview ranged from 20 to 35 min based on the interviewee’s mood, patience, and richness of experiences. Face-to-face interviews were held at places at which the participants were comfortable. Employees generally preferred the workplace while cancer patients and families were interviewed either in treatment centers or at their homes. All the interviews were conducted by a researcher with a Ph.D. in nursing and recorded by a voice recorder. The main questions, which varied according to the participant’s position, expertise, and knowledge, included "Would you please describe your views on the condition of home-based palliative care in Iran?", "How was your experience on home-based palliative care?", "How is it possible to integrate palliative care services into the PHC system for end-stage cancer patients?", "What are the challenges and opportunities?", and "How one can facilitate this process?". The researcher used probing questions to direct the interview towards the research objectives.

### Data analysis

Data analysis was performed simultaneously with holding the interviews based on the Lundman and Graneheim approach [17]. After each interview, the interviews were immediately transcribed verbatim and read carefully several times. The initial codes were extracted from the data and compared with each other to integrative related ones and form subcategories and categories based on their differences and similarities.

### Trustworthiness

Credibility, dependability, confirmability, and transferability were used according to Guba and Lincoln to ensure data rigor [18]. For acceptability, the participants were recruited in a way to obtain maximum diversity in terms of demographic variables such as age, gender, literacy level, income, and the type of cancer. After the initial analysis and extracting the primary codes, the participants were asked to confirm the accuracy of the codes and their interpretations. If the extracted codes were contradictory with the participants’ opinions, discussions and exchange of views were conducted to reach consensus on the initial codes. For transversality, the researchers tried to prepare an exact report of the details of the research process. For conformability, the researchers consulted with two faculty members who were experts in qualitative research to reach an agreement on the codes and their classifications.

### Ethical considerations

All the methods were performed in accordance with the relevant guidelines and regulations of the declaration of Helsinki (ethics approval and consent to participate). The aims and methods of the study were explained to all the participants, and necessary assurance was given to them for the anonymity and confidentiality of their information and audio files. Informed consent was obtained from all subjects. The participants had the right to withdraw during the study or at any other time. The Ethics Committee of Lorestan University of Medical Sciences approved the study protocol (ethical codes: LUMS.REC.1394.57 and IR.LUMS.REC.1398.217).

## Results

In this study, data were gathered through 22 face-to-face interviews with 21 participants (one of the participants was interviewed twice), as well as one focus group session (Tables 1 and 2). None of the participants don’t refused to participate in the study. Qualitative data analysis revealed three main categories and ten subcategories (Table 3).

**Table 1:** The characteristics of participants in individual interviews

Number	Age (years)	Degree of education	Work experience (years)	Position
1	62	Specialist	30	Active in policy making and home care provision
2	48	Specialist	25	Active in policy making and home care provision
3	38	Nurse	16	In charge of coordination of home-based palliative care services
4	52	Ph.D. in nursing	26	Lecturer of public health nursing
5	47	Nurse	19	In charge of the palliative care ward
6	46	Theologist	6	Religious expert
7	45	Nurse	16	Caregiver of home-based palliative care
8	43	Medical doctor	16	Palliative care physician
9	43	Social worker	11	Social worker
10	32	Employee	-	Caregiver
11	25	As assistant with a diploma	3	Assistant
12	34	General practitioner	4	Palliative care physician
13	53	Nursing assistant	8	Nursing assistant in the palliative care ward
14	33	Psychologist	3	Psychologist in home-based palliative care
15	29	Psychologist	5	Psychologist in home-based palliative care
16	40	Bachelor in nursing	17	Home care nurse
17	38	Bachelor in nursing	10	Home care nurse & in charge of a home care institute
18	38	Ph.D. in health policymaking	6	Faculty member in the field of health and health promotion
19	42	Ph.D. in epidemiology	17	Staff of the Health Deputy
20	37	Pharmacist	5	In charge of the drugs used in the network system
21	54	Employee	24	In charge of narcotics

**Table 2:** Participants of the focus group discussion

Number	Age (Years)	Level of education	Working experience (Years) Years	Position
1	62	Specialist	32	Active in home care provision
2	51	Specialist	18	Active in home care provision
3	48	Social medicine specialist	19	Representative of the Ministry of Health
4	47	Doctor	23	An agent of Iranian Health Insurance
5	54	Master degree in nursing	30	Manager of the home care institute
6	42	Bachelor degree in Nursing	16	A home care nurse
7	44	Social medicine specialist	8	Active in home care policymaking
8	42	Ph.D. in nursing	15	Active in home-based palliative medicine

**Table 3:** The categories and subcategories emerged from the data

Categories	Subcategories
The health system’s structure as an opportunity	Employing the network system for providing health services
	Establishment of a referral system
	Establishment of the family physician program and manpower diversity
Requirements	The position of home-care centers and their link with PHC
	Opioid use management
	Equipment management
	Financial support
	Legal issues
Outcomes	Facilitated access to services
	Good death

### The health system’s structure as an opportunity

Health care providers pointed out the capability of Iran’s health system to provide community-based health services to cancer patients. The participants acknowledged fundamental reforms in the structure of Iran’s health system after the launch of the network system, and the fact that people’s access to health services was facilitated by establishing comprehensive health service centers in residential areas. In the context of this system, patients can be referred from the first-level services to higher levels. Likewise, cancer patients can be provided with palliative care services in this structure. Three sub-categories were identified within this class, including employing the network system for providing health services, establishment of a referral system, and establishment of the family physician program and manpower diversity.

#### Employing the network system for providing health services

Experiences of the participants indicated that Iran’s network system possessed perfect opportunities to provide palliative care services to cancer patients. In this system, all members of the community have an electronic health record that is accessible to care providers in comprehensive health service centers. One of the experts stated: "…In health centers, the population is characterized and known…" [1]. Another participant mentioned: "…Cancer patients can receive community-based care in the context of PHC…" [18].

According to the participants’ experiences, a platform was launched in the context of comprehensive health centers in recent years, containing the health information of all people, to which caregivers can have access if needed. Therefore, known cases of cancer, as a member of the community, can be tracked and monitored using this platform. In this regard, one of the health experts noted: "…the integrated health system (i.e., SIB) has been launched in recent years, and it is possible to have access to all households’ health data…." [19]. The participants also admitted that within the context of this network, patients’ records can be transferred from the third and second levels to the first level of referral; this was stated by one of the participants: "… the SIB system should be linked to the patient’s information at hospitals, and if this is fulfilled, it would facilitate referrals and patient care programs…" [18].

#### Establishment of a referral system

According to the participants, the referral system is under the attention of health policymakers in current times, and good steps have been taken towards developing and completing such a system. Actually, it has been launched in villages and suburbs for several years now. As mentioned by the participants, providing care to cancer patients during end-life stages can be fulfilled using this referral system in a back-referral manner. Also, the participants clarified that even though the diagnosis of cancer is generally made in clinics and hospitals, the patient should be referred to the first level of the referral system in community-based health centers. One of the participants noted: "… When cancer is diagnosed in specialized centers [tertiary level of referral] … the patient should be referred to comprehensive health centers for further support [primary level of referral] …" [19].

The participants reiterated the positive experience of the back-referral system for the management of other diseases such as tuberculosis and leprosy, with patients being referred from higher specialized levels to comprehensive health care centers at the community level. A faculty member who was an expert of health policymaking mentioned: "…For example, patients with tuberculosis and leprosy are usually identified at a higher level by a specialist or a general physician, who then would refer patients to us [primary level of referral] … meaning a reverse direction…i.e., from a specialist or a subspecialist [tertiary level of referral] to the health center [primary level of referral] … " [18].

#### Establishment of the family physician program and manpower diversity

At the present time, the family physician program is running in rural and suburban areas in Iran and is also on the agenda of the Ministry of Health for large cities. So, the family physicians and care providers who are the members of the teams deployed to these areas can provide care for cancer patients at the end-stage of life. A participant said:"…Every family physician covers a limited population, so any person who needs palliative care can be under the coverage of one of these doctors…" [18].

Participants described that one of the potential elements that could be seen as an opportunity in the Iranian health system was the diversity of human resources at the community level and in comprehensive health centers. The manpower includes physicians, midwives/nurses, caregivers, psychologists, nutritionists, and other health experts who can be used to provide care for cancer patients at the community level. In this regard, one of the participants stated "…In addition, psychologists and public health experts can be used…" [18].

### Requirements

According to the experiences of the participants, necessary infrastructure should be available to be able to provide PHC-integrated palliative care. Data highlighted the lack of a characterized and clear link between home care centers and the PHC system, as well as a vague opioid use management approach, which was also true for equipment deliverance and social insurance support. All these can cause legal problems for palliative care provider centers. Under this category, five subcategories emerged, including the position of home care centers and their relationship with PHC, opioid use management, equipment management, financial support, and legal issues.

#### The position of home care centers and their relationship with PHC

Experiences of the participants showed that for the better use and more benefits of patients from home-based health services, several comprehensive health centers can be brought under the coverage of a single home-care provider team. The participants described the successful experience of the network system in providing maternity services tailored according to each population’s specifications and covered by several comprehensive health centers. On this issue, one of the participants stated: "…In this case, [for example] I recall maternity facilities and small surgery centers …places where a population of 20,000 is under the coverage of five to six health centers … on the natural flow of the population …. we launch maternity facilities in one of those health centers [we can follow the same pattern for providing care to cancer patients] …" [18].

#### Opioid use management

Among the problems of cancer patients during their last days are pain control and access to opioids. According to the participants, cancer patients monthly refer to the National Food and Drug Administration to receive their required drugs prescribed by a specialist. However, considering the capacity of the network system and the fact that health care providers can have access to patients’ files, it is amenable to provide patients with such medications from nearby comprehensive health care centers. In this regard, one of the pharmacists involved in drug distribution management said:"…Our main objective is to ensure easy, inexpensive, and rapid access to the medications… so we can deliver these medications to comprehensive health centers so that patients can have easy access to them…" [20].

#### Equipment management

Findings in this study highlighted appropriate equipment management as one of the most important requirements for providing home-based palliative care. As the participants acknowledged, consumables can be provided by patients and their families; on the other hand, health care providers can entrust them with non-consumable equipment. A home-care nurse stated: "…Suction devices can be portable …before transporting the patient to home, the patient’s bed and equipment must be prepared there …patients can be entrusted with these equipment…" [17]. Another health expert mentioned: "…In comprehensive health centers, we also need some other essential equipment such as resuscitation kits…"[19].

#### Financial support

According to the participants’ experiences, one of the most essential infrastructures for providing community-based palliative care is to determine the tariffs and insurance coverage so that patients can access these services without any barrier. Regarding that comprehensive health centers are integrated into the structure of the health system, their tariffs and franchises are pre-determined, and even some services in this structure are provided free of charge or at a cheap price. Likewise, palliative care services can be integrated into this structure, so that patients can benefit from these services by referring to and registering at comprehensive health centers in their places of residence. A participant noted: "…All the services provided are free of charge or are delivered at very cheap prices…within the network system, many diseases are covered by these services…" [19].

#### Legal issues

Data indicated the importance of addressing legal issues for patients, families, and caregivers during the provision of home-based palliative care. According to the experiences of the participants, these people should be aware of their own rights during the process and be committed to observe the rights of others. So, before the onset of the care provision process, families and comprehensive health centers should sign a contract, and all the services provided must be documented. In this regard, a participant said: "…The first thing that brings the center and the patient to feel committed to each other is to sign a contract…" [16].

### Outcomes

Data revealed that by integrating palliative care services into the network system, patients’ access to health services is facilitated, leading them to experience an easy and peaceful death. In this category, two sub-categories emerged, including facilitated access to services and easy death.

#### Facilitated access to services

According to the participants’ experiences, the integration of palliative care into the PHC system facilitates public access to such services. As mentioned by the participants, because these services are provided at a regional scale, access to them is easy for people. On the other hand, by the integration of these services into the network structure, their costs will be adjusted to the minimum as for other services provided in the system. In this regard, one of the participants mentioned:"…When a service is provided within the framework of the PHC structure, we expect it to be either free of charge or very cheap…this means affordable, which is in line with the UHC and WHO uphanded documents…. " [18].

#### Good death

According to the interviewees, a predetermined demise would facilitate the acceptance of death. During their last days, cancer patients are provided with the opportunity to reside at their homes and receive appropriate pharmaceutical and non-pharmacological treatments to reduce their physical and psychological complications so that they can have a better quality of life during this critical period. One of experts in this field underlined: "…patients and their family members should be endowed with peace…a psychologist can assist in improving the quality of death…" [18].

## Discussion

In this study, we aimed to explain stakeholders’ perceptions about the integration of palliative care with PHC. Our findings suggested that Iran’s health system was equipped with appropriate infrastructure to facilitate the integration of palliative care services into the PHC and the easy access of cancer patients to such services. However, there are a number of obstacles in this process that need to be reevaluated and revised so that families can have access to palliative care at their homes. This not only leads to the fulfilment of their care needs, but also provides the patient a peaceful death.

According to finding, the PHC is one of the pillars of the health system structure in Iran. Iranians can seek the health services required in each of the three levels of the referral system. In line with the findings of this study, WHO has urged the integration of palliative care aids with all other levels of the community-based services provided by the health system [13].

Our data showed that one of the opportunities provided by the structure of Iran’s health system was the establishment of the family physician program. In this regard, a team of several health care providers with different expertise in comprehensive health centers can deliver community-based palliative care, which of course, this process needs empowering these centers with new manpower and necessary training [19–22].

Our data highlighted the necessity of a close contact between home-based care provision centers and comprehensive health centers so that patients can use their support if needed. Nevertheless, in Iran, there is no systematic relationship between comprehensive health centers and home-based care providers at the present time [23]. Moreover, appropriate decisions should be taken to adjust the number of available comprehensive health centers covered by home care teams in order for them to be able to cover all patients and their health care requirements.

Findings showed that one of the issues that should be addressed in the process of care provision to cancer patients was easy access to opioids. In agreement with this notion, easy access to these medications was highlighted by another study as well [24]. In Iran, opioids are generally provided to patients with incurable diseases either at hospitals or by the national Food and Drug Administration only after prescription by a doctor [25]. According to upstream laws, health centers should provide a platform for delivering opioids to patients. It seems that there is a need for revising some laws related to the distribution of these drugs in Iran. During care provision to cancer patients, the equipment required by these patients, such as beds, mattresses, oxygen generators, etc., should be given special attention and provided along with consumables [7]. Comprehensive health centers are suggested to develop an independent unit in charge of equipment management and distribution and entrust patients with these items, which are returned after the patient’s demise.

Our data revealed that one of the factors that should be considered when providing care to cancer patients was the insurance coverage of the services provided, warranting the easy and cheap access of all cancer patients to palliative care [26]. As comprehensive health centers are governmental organizations affiliated with medical universities, their services should be covered by insurance companies and have pre-determined affordable prices.

Based on our findings, to provide community-based care services to cancer patients, the issues related to their medical rights should be addressed. According to the findings of another study, the process of providing PHC-integrated palliative care to cancer patients can face ethical challenges rooted in a shortage of resources, the lack of knowledge about palliative care, and the lack of support for the cases referred due to staff’s high workload [27]. Therefore, there is a prompt need for enacting new laws and developing appropriate guidelines based on these laws to ensure that the rights of patients, families, and palliative care providers are fulfilled and quality palliative care services are delivered to cancer patients.

An important outcome of this study is the improvement of the access of cancer patients and their families to health care services, which is in line with the goals of sustainable development and UHC programs [6]. Our data showed that by providing comprehensive care, including psychological and spiritual care, patients’ quality of death and families’ preparedness for the event can be improved. Consistently, the results of another study showed that the patients who died without receiving palliative care had more unmet needs [28]. Therefore, when providing community-based palliative care, all human aspects, including spiritual and psychological dimensions, should be considered. Moreover, the patient’s family must regularly receive psychological and spiritual support from the time of disease diagnosis until after the patient’s death.

Some of the limitations of this study include the following. Palliative care and home health service are new approaches of providing care in Iran, dating back to just the recent decade. This means limited access to experts, lack of infrastructure, and scarce centers providing these services, which are the most important limitations of this study. Other limitations included the scattering of experts throughout Iran, rendering communicating with and meeting them difficult. However, this problem was largely resolved by the efforts of the research team.

## Conclusion

Data analysis showed that the structure of Iran’s health system had the potential to provide palliative care to cancer patients in integration with PHC, raising numerous opportunities such as the establishment of the family physician program. Also, cancer patients can receive required services in the context of an integrated electronic health system by being referred from community-based specialized centers to comprehensive health centers around their places of residence (as the gateways of this reverse referral system). Beside these opportunities, there are also a number of obstacles for this process, such as the lack of instructions and guidelines, the lack of insurance coverage of palliative care services, and a shortage in competent human resources. These shortages must be addressed by implementing appropriate measures to finally be able to integrate palliative care services into the PHC system so that patients and their families can have easy access to such services at their homes. Consequently, patients can spend their last days beside their families and benefit from a good quality death with the preservation of human dignity along with the support of health care teams.

## Supplementary Information

**Additional file 1.** Raw data.

## Data Availability

The audio and text files of this qualitative study are available from the corresponding author if needed.

## Abbreviations

PHC: Primary Health Care
HSR: Health System Research
WHO: World Health Organization
UHC: Universal Health Coverage
SDG: Sustainable Development Goals
